# Viremia and Inflammatory Cytokines in Dengue: Interleukin-2 as a Biomarker of Infection, and Interferon-α and -γ as Markers of Primary versus Secondary Infection

**DOI:** 10.3390/pathogens12111362

**Published:** 2023-11-17

**Authors:** Thaís Bonato de Arruda, Lorena Bavia, Ana Luiza Pamplona Mosimann, Mateus Nobrega Aoki, Maria Lo Sarzi, Ivete Conchon-Costa, Pryscilla Fanini Wowk, Claudia Nunes Duarte dos Santos, Wander Rogério Pavanelli, Guilherme Ferreira Silveira, Juliano Bordignon

**Affiliations:** 1 Laboratório de Virologia Molecular, Instituto Carlos Chagas, Fiocruz, Curitiba 81350-010, Paraná, Brazil; thais.bonato9@gmail.com (T.B.d.A.); ana.mosimann@fiocruz.br (A.L.P.M.);; 2 Departamento de Biologia Celular, Setor de Ciências Biológicas, Universidade Federal do Paraná (UFPR), Curitiba 81531-980, Paraná, Brazil; 3 Laboratório de Ciências & Tecnologias Aplicadas a Saúde, Instituto Carlos Chagas, Fiocruz, Curitiba 81350-010, Paraná, Brazil; 4 Secretaria Municipal de Saúde de Cambé, Cambé 86057-970, Paraná, Brazil; 5 Laboratório de Protozoologia Experimental, Universidade Estadual de Londrina, Londrina 86057-970, Paraná, Brazilwanderpavanelli@yahoo.com.br (W.R.P.); 6 Instituto Carlos Chagas (ICC/Fiocruz-PR), Curitiba 81350-010, Paraná, Brazil

**Keywords:** *Dengue virus*, dengue acute phase, cytokines, IL-2, viremia

## Abstract

The pathogenesis of *Dengue virus* (DENV) infection is complex and involves viral replication that may trigger an inflammatory response leading to severe disease. Here, we investigated the correlation between viremia and cytokine levels in the serum of DENV-infected patients. Between 2013 and 2014, 138 patients with a diagnosis of acute-phase DENV infection and 22 patients with a non-dengue acute febrile illness (AFI) were enrolled. Through a focus-forming assay (FFU), we determined the viremia levels in DENV-infected patients and observed a peak in the first two days after the onset of symptoms. A higher level of viremia was observed in primary versus secondary DENV-infected patients. Furthermore, no correlation was observed between viremia and inflammatory cytokine levels in DENV-infected patients. Receiver operating characteristic (ROC) curve analysis revealed that IL-2 has the potential to act as a marker to distinguish dengue from other febrile illnesses and is positively correlated with Th1 cytokines. IFN-α and IFN-γ appear to be potential markers of primary versus secondary infection in DENV-infected patients, respectively. The results also indicate that viremia levels are not the main driving force behind inflammation in dengue and that cytokines could be used as infection biomarkers and for differentiation between primary versus secondary infection.

## 1. Introduction

Dengue is a febrile acute disease caused by one of the four *Dengue virus* (DENV) serotypes that are vectorially transmitted by *Aedes* sp. mosquitoes [1,2]. In 2013, it was estimated that there were more than 58 million symptomatic dengue cases worldwide, with more than 13,000 fatalities, at a global cost of USD 8.9 billion. Brazil alone accounted for USD 1.4 billion of that share [3,4]. In 2020, there were over 970,000 suspected dengue cases in Brazil, with an incidence of 466 cases/100,000 inhabitants [5]. During the first nine months of 2022, this number increased to over 2 million suspected dengue cases, representing 88% of the total number of cases in the Americas, with an incidence of more than 990 cases/100,000 inhabitants [6]. Between 2020 and 2022, more than 1600 patients died of dengue in Brazil [5,7,8].

According to the World Health Organization (WHO) guidelines, dengue is clinically classified as asymptomatic or causing mild symptoms (dengue without warning signs) or severe dengue. Patients with the mild form of dengue may experience fever, rash, headache, and pain. However, 3–7 days after illness onset, patients may experience warning signs, including persistent vomiting, severe abdominal pain, hyperventilation, bleeding gums, fatigue, restlessness, and blood in vomit [9]. If untreated, severe dengue progresses to fluid accumulation, respiratory distress, and shock, with a mortality rate of approximately 10% [9,10].

The mechanisms of disease progression are not fully understood. However, viral and host factors may play a role in the pathogenesis of the disease [11,12,13,14,15]. It is known that certain DENV strains can infect and replicate in host cells more efficiently, resulting in a high viral load [16,17,18]. The host immune response to DENV may be involved in the pathogenesis of severe dengue through a mechanism known as antibody-dependent enhancement (ADE), whereby antibodies generated during a previous DENV infection facilitate the cell entrance of the heterologous DENV serotype through an FcγR-mediated mechanism, increasing viremia [19,20].

Increased viral burdens can lead to a cytokine storm, whereby elevated levels of inflammatory cytokines are released [21,22]. Cytokine storms dysregulate the production of vasoactive mediators, leading to vascular permeability, plasma extravasation, and hemoconcentration, which are related to warning signs in severe dengue cases [10,22]. Furthermore, cytokine response patterns during DENV infection may also impact disease outcomes. A Th1 response, characterized by the production of cytokines such as IFN-γ, TNF-α, and IL-2, elicited a T-cell response and was associated with patients presenting mild disease. Nevertheless, a Th2 profile leads to the secretion of cytokines, such as IL-4, IL-10, and IL-13, which stimulate B cells to secrete antibodies and are more frequent in dengue patients presenting hemorrhagic disease [23,24].

Despite the potential association between disease severity, viremia, and inflammatory cytokine levels [11,25], no clear correlation has been found between these factors and disease progression. We therefore investigated the association between viremia and cytokine levels in the serum of acute-phase DENV-infected patients.

## 2. Materials and Methods

### 2.1. Patients

Between January 2013 and December 2014, patients who presented with clinical signs and symptoms of dengue infection in Cambé, Paraná State (southern Brazil) (23°16′33′′ S, 51°16′40′′ W) for up to seven days were invited to participate in the study. Exclusion criteria included acute febrile disease where the physician did not suspect dengue, the presence of symptoms of DENV infection lasting over seven days, or refusal to participate in the study. All patients were treated and clinically diagnosed in primary care units of the municipality of Cambé, and at the time of the first consultation, none needed hospitalization. This study was approved by the FIOCRUZ Research Ethics Committee (#617/11), and blood sampling and experiments were carried out following the guidelines and Brazilian regulations. All adult recruited patients provided written consent to participate in the study, and a parent or guardian provided informed consent on behalf of minors. Blood samples were collected by venipuncture and subsequently blind-coded to ensure patient anonymity. Blood samples were collected once and kept at 4 °C for up to 6 h until serum was separated by centrifugation and transferred to a new tube. The serum was kept at −20 °C for up to 24 h until transfer to a −80 °C. Patient clinical and epidemiological data were retrieved from the Information System for Notifiable Disease Forms, which was completed by the patients themselves (Sistema de Informação de Agravos de Notificação, SINAN).

Serum samples from suspected DENV-infected patients were tested for the presence of specific DENV antigens (NS1) or antibodies (IgM and IgG) using ELISA assay (PanBio, Windsor, Australia) according to the manufacturer’s protocol. Acute-phase DENV-infected patients presented NS1^+^/IgM^+^, NS1^+^/IgM^−^ or NS1^−^/IgM^+^ serological results, and the infection was later confirmed through virus isolation in C6/36 cells and serotyping using RT–PCR. Patients presenting compatible clinical signs of dengue with negative serological test results (NS1^−^/IgM^−^) were classified as non-dengue acute febrile illness patients (AFI). Furthermore, an IgG anti-DENV ELISA (Panbio, Windsor, Australia) was performed to categorize patients with secondary dengue infection. Patients presenting IgG^+^ against dengue during the first seven days after the onset of symptoms were considered to be suffering from a secondary infection, and patients who were IgG^−^ were considered to have a primary dengue infection [26]. A total of 138 patients with confirmed acute-phase DENV infection and 22 patients with acute febrile illness (AFI) who did not have a DENV infection, were enrolled in the study. Table 1 shows the demographic, serological, and clinical parameters of the patients.

### 2.2. Dengue Virus Isolation and Serotyping

Acute-phase serum samples from DENV-infected patients (NS1^+^/IgM^+^, NS1^+^/IgM^−^ or NS1^−^/IgM^+^) were tested for virus isolation in C6/36 *Aedes albopictus* cells (ATCC CRL-1660). An immunofluorescence assay was then performed using an anti-envelope protein (E) monoclonal antibody (4G2—ATCC HB-112) to confirm DENV infection. Briefly, the patient’s serum was diluted (1:10) in Leibovitz’s L-15 medium (Gibco, Grand Island, NY, USA) with 25 µg/mL gentamicin (Gibco, Grand Island, NY, USA) and 0.26% tryptose (Sigma, St Louis, MO, USA) and without fetal bovine serum (FBS). The patient’s serum (1:10; 500 µL) was then put through a 0.22 µm filter and inoculated in a T25 cell culture flask containing 2.0 × 10^6^ C6/36 cells. After 45–60 min, the inoculum was removed, and L-15 medium with 5% FBS (Gibco, Paisley, PA, UK) was added as previously described [27]. After 7–14 days or after the cytopathic effect was observed, C6/36 was detached, and the cells were stained for indirect immunofluorescence assay as previously described [27].

To determine the infecting serotype of the virus, RNA from the C6/36 cell culture supernatant was extracted using a QIAamp Viral RNA Mini Kit (QIAGEN, Hilden, NRW, Germany) as described by the manufacturer. The RNA was used in a one-step RT–PCR adapted protocol, which had previously been described [28]. Briefly, the RNA was mixed with one forward primer (D1) and four reverse primers (TS1, TS2, TS3, and TS4; for primer sequences, see Kuczera et al., 2017) [27] using a QIAGEN OneStep RT–PCR kit (QIAGEN, Hilden, NRW, Germany) as described by the manufacturer. The amplification cycle used was 50 °C for 30 min and 95 °C for 15 min, followed by 35 cycles of 95 °C for 30 s, 57 °C for 45 s, and 72 °C for 1 min, and a final extension cycle of 72 °C for 10 min. RNAs from DENV-1 (BR/90, GenBank: AF226685.2), DENV-2 (ICC/266), DENV-3 (BR/97-04, GenBank: EF629367.1), and DENV-4 (TVP/360, GenBank: KU513442) were used as controls for RT–PCR amplification.

### 2.3. Viremia Quantification in Serum Samples

Viremia in the serum of DENV-infected patients was quantified through viral titration in *Aedes albopictus* C6/36 cells using a focus-forming assay as previously described [29]. The C6/36 cells were cultured in L-15 supplemented with 5% FBS plus 0.26% tryptose and 25 µg/mL gentamicin at 28 °C. Briefly, 10-fold dilutions of patient serum (starting at 1:100) in L-15 media without FBS were used to inoculate C6/36 cells (1.0 × 10^5^ cells/well in 24-well plates). After 90 min of incubation at 28 °C, the inoculum was removed, a carboxymethylcellulose (CMC) (Sigma, St Louis, MO, USA) mixture (1:1 (*v*/*v*) of 3.2% CMC and L-15 media plus 10% FBS, 0.52% tryptose, and 50 µg/mL gentamicin) was overlaid on each well (500 µL/well), and the plates were incubated for seven days at 28 °C.

After incubation, the cells were fixed with 3% paraformaldehyde (PFA; Vetec, Rio de Janeiro, Brazil), permeabilized with 0.5% Triton X-100 (Sigma, St Louis, MO, USA), and stained using a mouse anti-flavivirus envelope monoclonal antibody (4G2) followed by an anti-mouse antibody conjugated to alkaline phosphatase (Promega, Madison, WI, USA). Detection was carried out using an NBT/BCIP substrate solution (Promega, Madison, WI, USA). The foci were counted (technical duplicates), and the virus titer was expressed as FFU_C6/36_/mL. Once the minimal serum dilution tested was 100-fold, the detection limit of this assay was 125 FFU_C6/36_/mL. Serum samples that did not produce any focus of infection on C6/36 cells but were positive on cell culture isolation and RT-PCR were considered 100 FFU_C6/36_/mL for calculation purposes.

### 2.4. Cytokine Determination in Serum Samples

The Th1/Th2/Th17 (IL-2, IL-4, IL-6, IL-10, IL-17A, IFN-γ, and TNF-α) and IFN-α cytokine (CBA Flex Set) and MCP-1 chemokine (CBA Fex Set) concentrations were measured in the serum of acute-phase dengue and AFI-infected patients in 2017 using a Cytometric Bead Array kit (BD Biosciences, San Diego, CA, USA) following the manufacturer’s protocol. The data were analyzed using FCAP Array v1.0.1 (Soft Flow, Pecs, Hungary) as indicated by the manufacturer. We used FlowJo v10 (Tree Star, Ashland, OR, USA) to identify the sample’s mean fluorescence and extract the detected curve points. The cytokine concentration was expressed in Log10 of pg/mL with the following limits of detection: IL-2, IL-4, IL-6, IL-10, IL-17A, IFN-γ, TNF-α (20–5000 pg/mL), IFN-α, and MCP-1 (10–2500 pg/mL). Cytokine values below the detection limit were calculated by FCAP array and FlowJo v10 and used in statistical analysis. If serum samples gave cytokine values above the limit of detection of the standard curve, the samples were diluted and measured again to fit in the interval of the standard curve.

### 2.5. Sequencing of the Envelope Gene and Phylogenetic Analysis

To determine the nucleotide sequence of the DENV envelope gene, RNA extracted from the C6/36 cell culture supernatant generated after virus isolation was used. Nine samples (five from 2013 and four from 2014) collected during the study were selected: LRV13/341 (GenBank accession number: OR179883), LRV13/350 (OR179882), LRV13/369 (OR179881), LRV13/412 (OR179884), LRV13/467 (OR179887), LRV14/893 (OR179885), LRV14/993 (OR179889), LRV14/1139 (OR179888), and LRV14/1166 (OR179886). The complete E sequence of each sample was determined and used to confirm the DENV serotype and genotype.

Viral RNA was extracted using the QIAamp Viral RNA Mini kit (QIAGEN, Hilden, NRW, Germany) per the manufacturer’s instructions. The RNA was reverse transcribed using random primers (100 pmol/µL) and the ImProm-II Reverse Transcription System (Promega). The E gene was amplified using a high-fidelity enzyme (LongRange PCR, Qiagen), as well as the forward primer M5′ (5′-GCGGATCCTCCGTGGCACTGGCCCCACAC-3′) and reverse primer CN12 (5′-GCATCTCCTACAACCACTG-3′), both at 0.4 µM. Amplification was performed in a GeneAmp PCR System 9700 thermocycler (Thermo Fisher Scientific) using the following cycling conditions: 93 °C for 3 min, followed by 35 cycles at 93 °C for 15 s, 50 °C for 30 s, and 68 °C for 2 min. The samples were then kept at 4 °C until analysis in a 0.8% agarose gel. The PCR product of the expected size (1997 bp) was purified using a High Pure PCR kit (Roche Life Science). Sanger sequencing of the purified PCR products was performed at Macrogen (Seoul, Korea) using the following primers: M5′ (5′-GCGGATCCTCCGTGGCACTGGCCCCACAC-3′), PhD17 (5′-GGGATCTTGCATGGTGC-3′), D1Domb-(5′-TGCGGTACCGCTGCTTCCTTTCTTGAACCA-3′), D1Domb+ (5′-ACGGGATCCGTGATGTGCACAGGCTCATTT-3′), and CN12 (5′-GCATCTCCTACAACCACTG-3′).

The consensus sequence was assembled using the Phred/Phrap/Consed package (www.phrap.org; accessed on 19 March 2017) [30,31,32]. The sequences were then aligned, and nucleotide and amino acid changes were verified using BioEdit v. 7.0.5 [33] for Windows. Using E sequence analysis, samples LRV13/467, LRV14/993, and LRV14/1139 were excluded because they were identical to other sequences (LRV13/467 = LRV14/369, and LRV14/993 = LRV14/1139 = LRV14/1166). The remaining six sequences from the E gene and other sequences from dengue serotype-1 from NCBI were used to generate a dataset with a total of 160 sequences. Those sequences were aligned using ClustalW as implemented in BioEdit v 7.0.5, and the phylogenetic tree was inferred through maximum likelihood using PhyML 3.0, with a GTR+G+I model with a bootstrap of 1000 replicates. A dengue serotype 3 sample (NC_001475) was used to root the three. Some of the clades with support inferior to 10 were collapsed for better image resolution, hiding 88 DENV-1 strains (Supplementary Table S1). Strains were identified according to GenBank accession number/country/year. Lineage classification was based on Bruycker-Nogueira et al. (2015) [34].

### 2.6. Statistical Analysis

**Figure 1:**
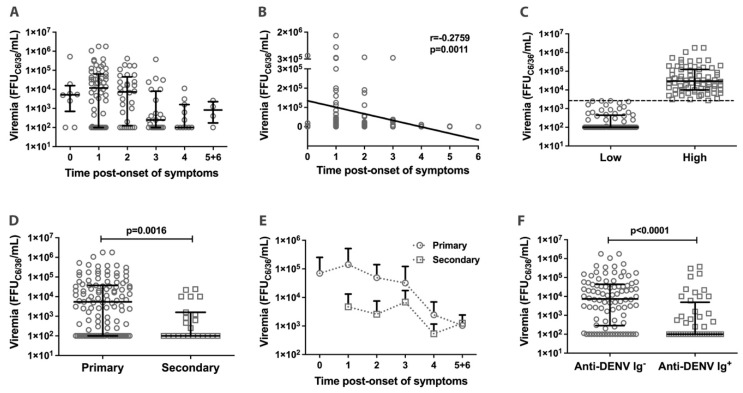
Viremia profile in acute phase dengue patients. (**A**) Distribution of viremia over time of infection (n = 138). (**B**) Correlation between viremia and time of infection (n = 138). (**C**) Viremia classification as low (circles; n = 68) and high (squares; n = 70), considering the median (2688 FFU_C6/36_/mL) represented by the dotted line. (**D**) Viremia among patients with primary (IgG^−^; circles; n = 115) and secondary (IgG^+^; squares; n = 23) dengue infection based on the presence of anti-dengue IgG^+^ in serum samples until the seventh day after the onset of symptoms. (**E**) Viremia of patients with primary (IgG^−^; circles; n = 115) and secondary (IgG^+^; squares; n = 23) dengue infection according to the day of sample collection post-onset of symptoms. (**F**) Viremia among acute-phase dengue patients with (Anti-DENV Ig^+^/IgM and/or IgG; squares; n = 44) and without (Anti-DENV Ig^−^/IgM and/or IgG Anti-DENV; circles; n = 94) immunoglobulins against dengue. Data are presented as the median and interquartile range except for Figure 1E, which represents the mean plus SD. Significant differences were considered when *p* < 0.05.

Normal distributions of the continuous variables were tested using the Kolmogorov–Smirnov method. Categorical data are presented as counts and percentages, and continuous data are expressed as medians and interquartile ranges, with exceptions described in the caption for Figure 1 and Table 1. Fisher’s exact and chi-square tests were used in the bivariate analysis of categorical variables, such as gender, serology, and clinical and hemorrhagic symptoms. The Mann–Whitney U test was used to compare medians for continuous variables. Spearman’s rank correlation coefficient was used to investigate the relationship between cytokine levels. A *p* value < 0.05 was considered significant. ROC curve analysis and the Youden index (J = sensitivity + specificity − 1) were used to find the optimal threshold point from the ROC curve to discriminate between dengue and AFI patient states considering cytokine levels [35,36].

## 3. Results

### 3.1. Patients with Dengue virus and Laboratory Diagnosis

In a recent epidemiological study, we investigated the correlation between dengue and poverty in southern Brazil, in addition to the climatic characteristics of the region, especially precipitation and temperature [37]. We increased the sample collection from this previous study with the aim of analyzing the correlation between viremia and inflammatory cytokine/chemokine levels in DENV-infected patients. Thus, between 2013 and 2014, a total of 138 DENV-infected patients in the city of Cambé, southern Brazil, were enrolled in the study (Table 1). Serum samples from 22 AFI-infected patients, also from Cambé, were included in the analysis as controls (Table 1). All 138 dengue samples were either positive for NS1^+^/IgM^−^, NS1^−^/IgM^+^, or NS1^+^/IgM^+^, confirming that they were acute-phase DENV infections. The 22 AFI patients were negative for the NS1 antigen and anti-dengue IgM antibody (Table 1). No difference was observed in demographic and clinical parameters for either DENV- or AFI-infected patients (Table 1).

The presence of specific anti-DENV IgG antibodies has been used to discriminate between primary and secondary dengue infections [26]. Thus, once all serum samples were collected up to the seventh day after the onset of symptoms, the absence or presence of anti-dengue IgG antibodies was used to classify patients with primary or secondary DENV infection, respectively [26]. Of the 138 samples, 115 were classified as primary DENV-infected patients, and 23 were classified as secondary DENV-infected patients (Table 1).

Furthermore, all 138 serum samples were confirmed as acute-phase dengue through virus isolation in C6/36 cell culture followed by immunofluorescence as previously described (Supplementary Figure S1) [27]. A one-step RT–PCR on C6/36 cell culture supernatant identified 137 samples as DENV serotype 1 and one as DENV serotype 4 (Supplementary Figure S2) [27]. The nucleotide sequence of the envelope gene from nine DENV-1 samples confirmed the presence of genotype V and lineages 1b and 2 (Supplementary Figure S3).

### 3.2. Dengue Viremia in Acute Phase Patients

The pathogenesis of severe dengue infection usually correlates with an enhancement of infection (ADE) and DENV replication, leading to higher viremia and the secretion of high levels of inflammatory cytokines (cytokine storm) [11,12,21,22]. Thus, after confirming acute DENV infection in 138 patients, viremia was quantified in serum samples (Figure 1A). The results indicated a weak negative correlation between viremia and the days since the onset of symptoms (*p* = 0.0011 and r = −0.2759; Figure 1B).

The median viremia obtained for all 138 patients with DENV was 2688 FFU_C6/36_/mL. This number was used to classify the samples as either having high (≥2688 FFU_C6/36_/mL; median 29,625 FFU_C6/36_/mL; n = 70) or low (<2688 FFU_C6/36_/mL; median 100 FFU_C6/36_/mL; n = 68) viremia (Figure 1C). We also compared the viremia levels in primary versus secondary DENV infections based on the presence of anti-dengue IgG antibodies before day 7 after the onset of symptoms (Figure 1D,E). Viremia levels were higher in primary DENV-infected patients (median of 5400 FFU_C6/36_/mL; n = 115) than in secondary DENV-infected patients (median of 100 FFU_C6/36_/mL; n = 23) (*p* = 0.0016; Figure 1D). This difference persisted independently of the sample collection day after the onset of symptoms (Figure 1E). Finally, the presence of specific anti-DENV antibodies (IgM and/or IgG) correlated with a reduction in viremia compared to samples positive for NS1 antigen but negative for the specific antibodies (*p* < 0.0001; Figure 1F).

### 3.3. Inflammatory Cytokines in DV Patients

**Figure 2:**
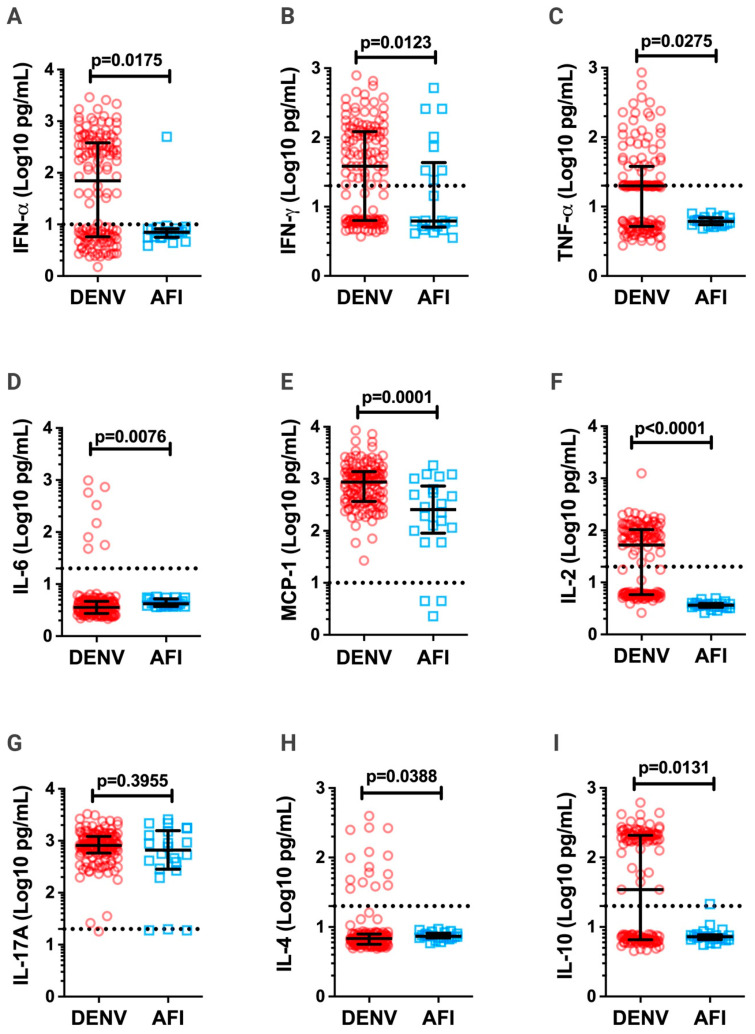
Serum cytokine/chemokine levels in dengue (DENV) and acute febrile illness (AFI) patients. The concentrations of cytokines/chemokines IFN-α (**A**), IFN-γ (**B**), TNF-α (**C**), IL-6 (**D**), MCP-1 (**E**), IL-2 (**F**), IL-17A (**G**), IL-4 (**H**), and IL-10 (**I**) were analyzed in the serum of acute-phase DENV-infected patients (red circles; n = 138) and AFI-infected patients (blue squares; n = 22), non-DENV-infected. Cytokines/chemokines were measured using the cytometric bead array (CBA) technique by flow cytometry (FACS Canto II). Cytokine/chemokine values are expressed in Log10 (pg/mL). All data are presented as the median and interquartile range. Significant differences were considered when *p* < 0.05. Dashed lines in each graph represent the detection limit of the CBA assay.

The role played by inflammatory cytokines in triggering the endothelial damage observed in severe dengue cases is well established [13,20,21,25,38,39]. Therefore, we compared the levels of IL-2, IL-4, IL-6, IL-10, IL-17A, IFN-γ, TNF-α, IFN-α and MCP-1 in the serum of acute-phase DENV-infected patients versus AFI, non-DENV-infected patients. Except for IL-17A, all cytokines/chemokines had higher expression levels in DENV-infected patients than in AFI patients (Figure 2).

ROC curve analysis, which uses the area under the curve (AUC), indicated that IL-2 and MCP-1 presented the best combined sensitivity and specificity to differentiate DENV-infected from AFI patients (Supplementary Figure S4; Table 2). However, using the Youden index, which measures the effectiveness of a threshold value for a marker [40], MCP-1 was less efficient in differentiating between DENV-infected versus AFI patients (cutoff < 192.7 pg/mL, 45.45% sensitivity, and 94.93% specificity; Table 2). This index also confirmed that IL-2 could be used to differentiate between DENV-infected and AFI patients (cutoff < 4.975 pg/mL, 100% sensitivity and 94.93% specificity; Table 2).

Once there is a dichotomy between a Th1 and Th2 response in DENV infection [41,42,43], a correlation analysis between IL-2 and cytokines from Th1, Th2, Th17, and the chemokine MCP-1 was performed. The results showed a positive correlation between IL-2 and IFN-α, IFN-γ, TNF-α, IL-17A, and IL-10, and a negative correlation with IL-4 and IL-6 (Table 3). These data indicate that for acute-phase DENV-infected patients, there was a trend to develop a Th1-like phenotype that correlates with a disease without warning signs or hemorrhage (Table 1 and Table 3) [44,45].

### 3.4. Correlation between Viremia and Inflammatory Cytokines

**Figure 3:**
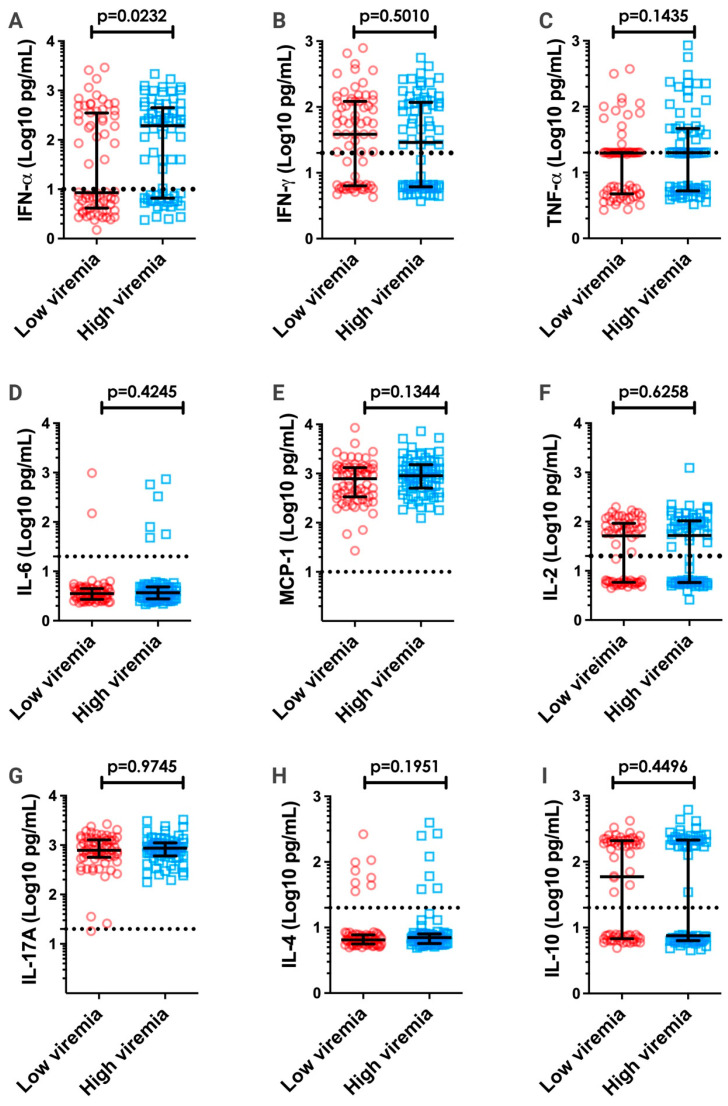
Serum cytokine/chemokine levels in dengue patients presenting low and high viremia. The cytokine/chemokine levels of IFN-α (**A**), IFN-γ (**B**), TNF-α (**C**), IL-6 (**D**), MCP-1 (**E**), IL-2 (**F**), IL-17A (**G**), IL-4 (**H**), and IL-10 (**I**) were categorized based on low (red circles; n = 68) and high (blue squares; n = 70) viremia based on the median value for all DENV samples (2688 FFU_C6/36_/mL). Cytokine/chemokine values are expressed in Log10 (pg/mL). All data are presented as the median and interquartile range. Significant differences were considered when *p* < 0.05. Dashed lines in each graph represent the detection limit of the CBA assay.

Several studies have suggested that viremia levels may play a major role in the pathogenesis of dengue, leading to more severe disease and the secretion of inflammatory cytokines [46,47,48,49]. However, the association between inflammation and viremia levels in DENV-infected patients remains controversial [11,12,25]. A comparison was therefore made between the levels of inflammatory cytokines and viremia (Figure 3). The data indicated that patients with high viremia levels had higher levels of IFN-α. Spearman’s correlation test was performed to evaluate a possible correlation between overall viremia levels and measured cytokines/chemokines; however, no significant correlation was observed (Supplementary Table S2).

**Figure 4:**
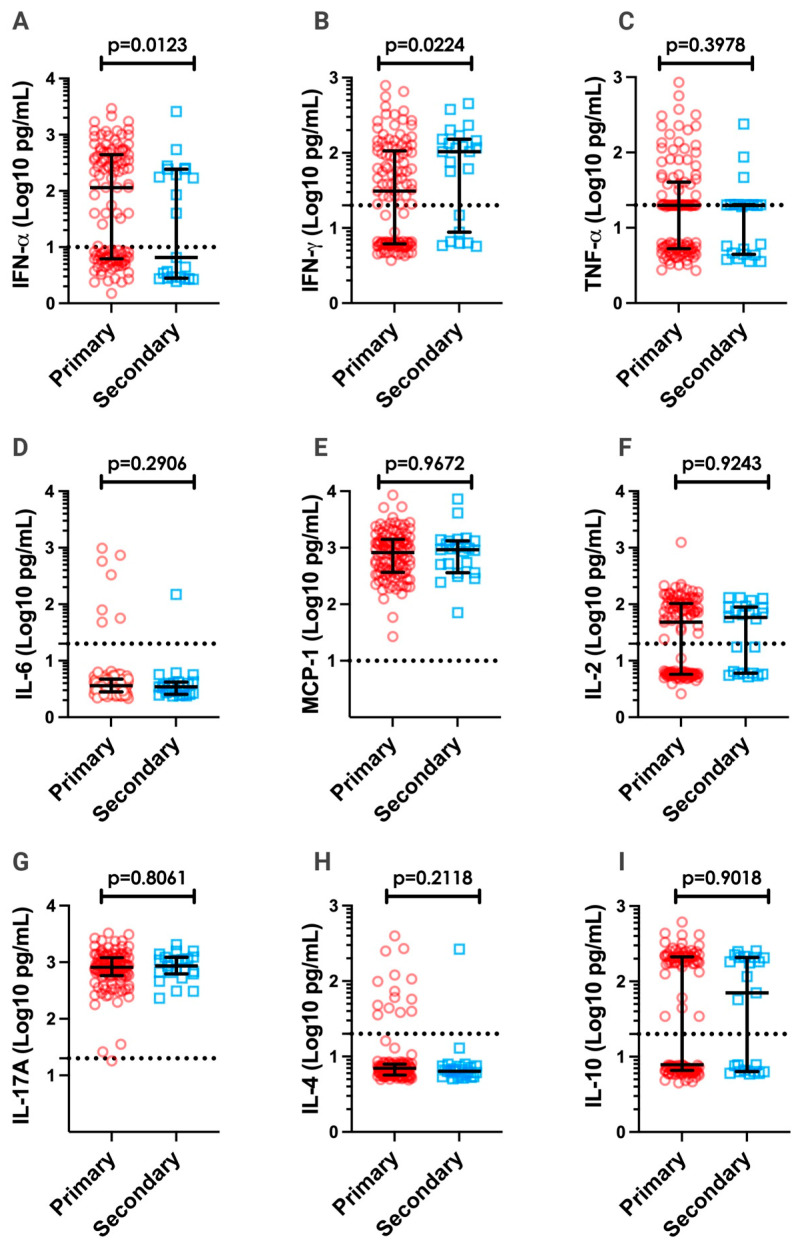
Serum cytokine/chemokine levels in primary and secondary dengue-infected patients. The cytokine/chemokine levels of IFN-α (**A**), IFN-γ (**B**), TNF-α (**C**), IL-6 (**D**), MCP-1 (**E**), IL-2 (**F**), IL-17A (**G**), IL-4 (**H**), and IL-10 (**I**) were categorized based on primary (red circles; IgG^−^; n = 115) and secondary (blue squares; IgG^+^; n = 23) dengue-infected patients (NS1 and/or IgM positive samples). Cytokine/chemokine values are expressed in Log10 (pg/mL). All data are presented as the median and interquartile range. Significant differences were considered when *p* < 0.05. Dashed lines in each graph represent the detection limit of the CBA assay.

Secondary heterologous infection has been proposed as one of the mechanisms responsible for severe dengue disease [19,20]. Thus, we compared cytokine/chemokine secretion in primary versus secondary DENV-infected patients, as cytokine storms occur more frequently in the latter cases. The results indicate that primary infection with dengue resulted in higher IFN-α levels than secondary infection (Figure 4A). However, higher IFN-γ levels were observed in secondary-infected patients than in primary-infected patients (Figure 4B). For the other measured cytokines/chemokines (IL-2, IL-4, IL-10, IL-17A, MCP-1, and TNF-α), no difference was observed between primary and secondary DENV-infected patients (Figure 4C–I).

## 4. Discussion

A wide range of clinical presentations is associated with DENV-infected patients, ranging from asymptomatic or oligosymptomatic cases to severe infection with hemorrhagic manifestations [10]. The pathogenesis of the infection depends on host and viral factors, such as the presence of preexisting antibodies and mutations in the viral genome that lead to better replication or inhibition of the type I IFN response [50,51,52]. Given the complexity of dengue immunopathology, the identification of infection or disease severity biomarkers is important for clinicians.

We analyzed the levels of viremia and inflammatory cytokines in DENV-infected and AFI patients. Clinical parameters revealed that both populations presented with similar symptoms, demonstrating the challenges in distinguishing dengue from other febrile diseases. This is mentioned in the WHO’s dengue guidelines, which emphasize that the diagnosis of dengue infection needs confirmation using specific laboratory techniques, such as ELISA and RT–PCR [10]. Here, it was shown that IL-2 is a potential marker to differentiate between DENV- and AFI-infected patients in southern Brazil. However, a study performed in Taiwan indicated IL-10 as a marker of dengue infection [53], which demonstrates the complexity of finding a definitive biomarker for DENV infection. Additionally, the data presented here indicate that the viremia levels in DENV-infected patients are high in the first days after symptom onset and drop off quickly. No correlation was observed between the levels of viremia and the tested inflammatory cytokines.

Viremia levels in patients with dengue result from a balance between the strain’s ability to infect and replicate in the host and the immune system’s capacity to control replication [52,54]. Here, following the detection of the viral particle, the viremia levels of DENV-infected patients were determined using a focus-forming assay in C6/36 cells, which measures viable virus particles. We observed high viremia levels in the first two days after the onset of symptoms, with a reduction occurring over the following days. The short duration of viremia levels in dengue has already been demonstrated, and blood DENV levels are significantly reduced 3–6 days after the onset of symptoms [55].

Furthermore, secondary dengue infections by a heterologous serotype have been reported as a significant risk factor in more than 97% of severe dengue cases [55]. Due to the ADE mechanism in secondary heterologous infections, higher viremia levels may be expected in these patients. However, we demonstrated that viremia levels were higher in patients with a primary rather than secondary infection. This finding had already been demonstrated and could be a serotype-specific phenomenon or associated with the appearance of specific anti-dengue antibodies, both IgM and IgG, which emerge faster in secondary infections [26,54,55]. Additionally, more hemorrhagic cases of DENV in primary infection than in secondary infection, independent of viremia, have been reported in Chinese DENV-infected patients [56]. In contrast, a recent study in Vietnamese DENV-patients found evidence that higher viremia increases the risk of severe disease independently of the virus serotype or patient immune status [57]. Thus, there is still no consensus in the literature about whether viremia levels in DENV patients are definitely related to immunopathogenesis and disease severity [11,58,59].

Secondary DENV infections are associated with increased production of inflammatory cytokines and the development of cytokine storms [45,50,59]. Using the AG129 (IFNAR and IFNGR KO) animal model, a peak of inflammatory cytokines (IL-6, TNF-α, MCP-1, IL-12p70, and IFN-γ) was observed in the serum of mice 1–2 days after virus levels had reached their maximum [53]. Additionally, the efficient virus replication control with antivirals reduces the levels of inflammatory cytokines in the sera of mice, indicating that a close relationship may exist between viremia levels and inflammation in mice [53].

We showed that IFN-α, IFN-γ, TNF-α, IL-6, MCP-1, IL-2, IL-4, and IL-10 production was higher in patients with dengue than in those with AFI. Furthermore, the production of the antiviral cytokine IFN-α was higher in patients with high viremia levels. Similar results were found in Chinese DENV patients; however, a negative correlation between IFN-α levels and platelet counts was observed, suggesting that dengue severity is affected by both viral and immune factors [56]. A balance between the antiviral activity of type I IFN and DENV countermeasures to block the type I IFN response could impact viremia levels observed in DENV-infected patients [51]. However, it has been shown that the IFN-α levels did not differ between Thai children with dengue hemorrhagic fever (DHF) and those with dengue fever (DF), which indicates that despite its importance for controlling DENV replication, IFN-α levels do not impact dengue pathogenesis [60]. Meanwhile, higher IFN-γ levels were observed in patients with secondary infection than in those with primary infection. High IFN-γ levels have been detected in patients with dengue, which is also associated with severe disease [12,61]. Similarly, IFN-γ produced by T cells stimulates mononuclear cells to secrete TNF-α, tissue factor, and platelet-activating factor, which are associated with severe disease. IFN-γ has already been associated with secondary heterologous infection [62,63].

Both IFN-α and IFN-γ are relevant restriction factors for DENV infection, and animals lacking their receptors are highly susceptible to infection [39]. It has been shown that T cells collected from children who developed subclinical secondary infection produced more TNF-α, IFN-γ, and IL-2 after stimulation with dengue antigens than those from children presenting symptomatic secondary DENV infection [64]. CD4^+^ and CD8^+^ T cells from dengue-infected patients stimulated with envelope and NS3 dengue antigens produce multifunctional T cells secreting IFN-γ, TNF-α, and IL-10. The number of multifunctional T cells is higher in patients with mild dengue disease than in patients with warning signs. Ultimately, the number of multifunctional T cells correlates with the platelet nadir in DENV-infected patients [65].

We also found that DENV-infected patients had higher levels of IL-2 than AFI patients without dengue, which supports the findings from a previous study [66]. IL-2 is a T-cell growth factor that acts as a mitogen to increase T-cell proliferation [67,68,69]. It is indirectly needed to generate TGF-β-mediated CD4^+^/CD25^+^/Foxp3^+^ T regulatory cells [70]. Thus, IL-2 secretion is crucial in dengue-infected patients for the differentiation of naïve CD4^+^ T cells into regulatory CD4^+^/CD25^high^/Foxp3^+^ T cells [71]. T CD4^+^ regulatory cells control the production of vasoactive cytokines after dengue stimulation, and a high ratio of T regulatory cells/effector T cells is associated with mild disease [71]. The production of IL-2 after dengue infection seems to be beneficial and is associated with a better clinical outcome. IL-2 modulates the expression of cytokine and transcription factor receptors that differentiate T helper cells into both Th1 and Th2 cells [72]. However, we found that a positive correlation may exist between IL-2 and Th1 cytokines in DENV-infected patients. Some authors have indicated that a Th1 CD4^+^ profile is protective against dengue, while a shift to Th2 is involved in disease severity [41,42,43]. A Th2 cytokine profile was observed in Thailand DENV patients presenting high viremia and plasma leakage, which was associated with higher levels of IL-6 and IL-8 at the early phase of disease [73].

Although the study addresses a population that was, until recently (two decades ago), mostly naïve for dengue infection, the study presents limitations that need to be considered. The study was performed in a single site (Cambé, Southern Brazil) and restricted to primary care-based DENV-infected patients. Dengue patients were predominantly infected with serotype 1, which makes a comparison to other dengue serotypes impossible. A small number of dengue-infected and control (AFI-patients) samples were analyzed in the study, and the lack of a specific diagnosis for AFI-patients makes it difficult to establish comparisons between dengue and other specific acute febrile illnesses. Another point is the fact that we only analyzed non-severe dengue patients, which makes it difficult to confirm how the cytokine/chemokine levels presented here impact in the evolution of the disease to a severe disease. Also, a longitudinal analysis would raise more comprehensive data on relation between viremia and inflammation. Finally, it is important to point out that serum samples were collected in primary-care units, kept at 4 °C (for up to 6 h) and −20 °C (for up to 24 h), then transferred to −80 °C, where samples were kept for thee-to-four years before cytokines/chemokines were measured. Thus, although the literature shows that the samples are stable under these conditions [74], the degradation of some cytokines cannot be excluded.

The role of viremia levels in the immunopathogenesis of dengue is still a matter of discussion in the literature [11,54,56]. Furthermore, differences in the results between studies could be explained by different virus subtypes or by differences in sequential dengue infection between studies [26]. Despite the study limitations, it is important to mention that the main objective of this study was to investigate whether dengue viremia would have some impact on cytokine/chemokine levels in serum. The data reinforce the concept that viremia is not the main trigger leading to inflammation on dengue infection. Also, it is possible to strengthen the association between IL-2 and the Th1 profile results in a milder form of the disease and that IFN-α and IFN-γ could be used as biomarkers for primary versus secondary infection in DENV-infected patients. Finally, the presented data highlight the importance of conducting comprehensive longitudinal studies that look at infection in different populations (children versus adults), affected by different virus serotypes, primary versus secondary infection, and mild versus severe dengue cases to provide a better idea of the role of viremia levels in the pathogenesis of dengue.

## 5. Conclusions

Based on our analysis of primary-care acute-phase DENV-infected patients, we propose that viremia is not the main inflammation trigger after dengue infection. Our results reinforce previous findings that the Th1 cytokine profile is associated with mild dengue. Finally, based on the ROC curve analysis, we suggest that IL-2 may be a potential biomarker of DENV infection and that IFN-α and IFN-γ may be potential markers for primary versus secondary infection in DENV-infected patients.

## Figures and Tables

**Table 1:** Demographic, serological, and clinical parameters of dengue and febrile illness patients enrolled in the study.

Parameters		DENV	AFI	*p*-Value
**Patients**		138 (100%)	22 (100%)	-
Age (years) mean ± SD		35.26 ± 17.52	28.45 ± 16.07	0.0603
Gender	Female	68 (49.3%)	14 (63.6%)	0.2545
	Male	70 (50.7%)	8 (36.4%)	
Serology	NS1^+^/IgM^−^	105 (76.1%)	0	-
	NS1^−^/IgM^+^	9 (6.5%)	0	
	NS1^+^/IgM^+^	24 (17.4%)	0	
	IgG^+^	23 (16.7%)	5 (22.7%)	0.5455
	IgG^−^	115 (83.3%)	17 (77.3%)	
Clinical	Fever	124 (89.8%)	18 (81.8%)	0.4697
Symptoms	Headache	116 (84.1%)	19 (86.4%)	
	Myalgia	106 (76.8%)	16 (72.7%)	
	Prostration	90 (65.2%)	9 (40.9%)	
	Retro-orbital pain	70 (50.7%)	12 (54.5%)	
	Nausea	73 (52.9%)	11 (50%)	
	Arthralgia	56 (40.6%)	11 (50%)	
	Diarrhea	14 (10.1%)	6 (27.2%)	
	Exanthema	9 (6.5%)	0	
	Others *	9 (6.5%)	2 (9.1%)	
Hemorrhagic	Tourniquet test	6 (4.3%)	2 (9.1%)	1.0000
Symptoms	Petechiae	2 (1.4%)	0	
	Epistaxis	0	0	
	Gum bleeding	0	0	
	Gut bleeding	0	0	

* Others: abdominal pain, lower back pain, lethargy, skin pallor, dizziness, chills, pruritis. DENV—*Dengue virus*; AFI—acute febrile illness patients.

**Table 2:** Area under the curve and Youden values for the calculation of sensitivity and specificity of each cytokine as a marker for *Dengue virus* infection.

Cytokine	AUC	Youden Index	Cut off	Sensitivity%	Specificity%
IFN-α	0.6581	0.5197	<9.930	95.45	56.52
IFN-γ	0.6665	0.3445	<6.300	59.09	75.36
TNF-α	0.6467	0.5797	<13.61	100.0	57.97
IL-6	0.6775	0.5145	>3.615	100.0	51.45
MCP-1	0.7546	0.4038	<192.7	45.45	94.93
IL-2	0.9890	0.9493	<4.975	100.0	94.93
IL-17A	0.5567	0.2754	<562.8	50.00	77.54
IL-4	0.6375	0.3399	>6.890	81.82	52.17
IL-10	0.6650	0.5072	<27.90	100.0	50.72

**Table 3:** Correlation between IL-2 serum concentration and the other cytokines/chemokines in DENV-infected patients.

IL-2 vs	Spearman (r)	95% Confidence Interval	*p*-Value
IFN-α	0.3225	0.1594 to 0.4685	0.0001
IFN-γ	0.3516	0.1912 to 0.4938	<0.0001
TNF-α	0.5200	0.3822 to 0.6352	<0.0001
IL-6	−0.4963	−0.6157 to −0.3545	<0.0001
IL-4	−0.2256	−0.3827 to −0.05574	0.0078
IL-10	0.7322	0.6410 to 0.8031	<0.0001
IL-17A	0.2137	0.04330 to 0.3720	0.0118
MCP-1	0.06995	−0.1033 to 0.2391	0.4150

## Data Availability

The data presented in this study are available on request from the corresponding authors. The obtained nucleotide sequences were deposited in GenBank under the following accession numbers OR179883, OR179882, OR179881, OR179884, OR179887, OR179885, OR179889, OR179888 and OR179886.

## Supplementary Materials

The supporting information can be downloaded at https://www.mdpi.com/article/10.3390/pathogens12111362/s1: Figure S1: Representative images of immunofluorescence on C6/36 cells to confirm dengue virus diagnosis in serum samples; Figure S2: Representative agarose gel of one-step RT-PCR to confirm dengue virus diagnosis/serotype in serum samples; Figure S3: Phylogenetic analysis of the E coding region of *Dengue virus* strains from Cambé; Figure S4: Area under the curve (AUC) analysis of cytokines/chemokines in acute phase dengue patients; Table S1; Table S2: Correlation between viremia and cytokine/chemokine levels in the serum of DENV-infected patients.
