# Supplementary material for: Viremia and Inflammatory Cytokines in Dengue: Interleukin-2 as a Biomarker of Infection, and Interferon-α and -γ as Markers of Primary versus Secondary Infection
Source: Pathogens. 2023 Nov 17;12(11):1362. doi: 10.3390/pathogens12111362 (PMC10675515; doi:10.3390/pathogens12111362)
Supplement: Supplementary file 1 [file pathogens-12-01362-s001.zip › pathogens-2614882-Supplementary Material.pdf]

Supplementary Material

Viremia and inflammatory cytokines in dengue: interleukin-2 as a biomarker of infection, and Interferon-α and -γ as markers of primary *versus* secondary infection

Thaís Bonato de Arruda[1†], Lorena Bavia[1,2†], Ana Luiza Pamplona Mosimann[1&], Mateus Nobrega Aoki[1,3], Maria Lo Sarzi[4], Ivete Conchon-Costa[5], Pryscilla Fanini Wowk[1&], Claudia Nunes Duarte dos Santos[1], Wander Rogério Pavanelli[5#], Guilherme Ferreira Silveira[1#], Juliano Bordignon[1#&]

[1]Laboratório de Virologia Molecular, Instituto Carlos Chagas, Fiocruz, Curitiba, 81350-010, Paraná, Brazil;
[2]Departamento de Biologia Celular, Setor de Ciências Biológicas, Universidade Federal do Paraná (UFPR), Curitiba, 81531-980, Paraná, Brazil;
[3]Laboratório de Ciências & Tecnologias Aplicadas a Saúde, Instituto Carlos Chagas, Fiocruz, Curitiba, 81350-010, Paraná, Brazil;
[4]Secretaria Municipal de Saúde de Cambé, Cambé, 86057-970, Paraná, Brazil;
[5]Laboratório de Protozoologia Experimental, Universidade Estadual de Londrina, Londrina, 86057-970, Paraná, Brazil;

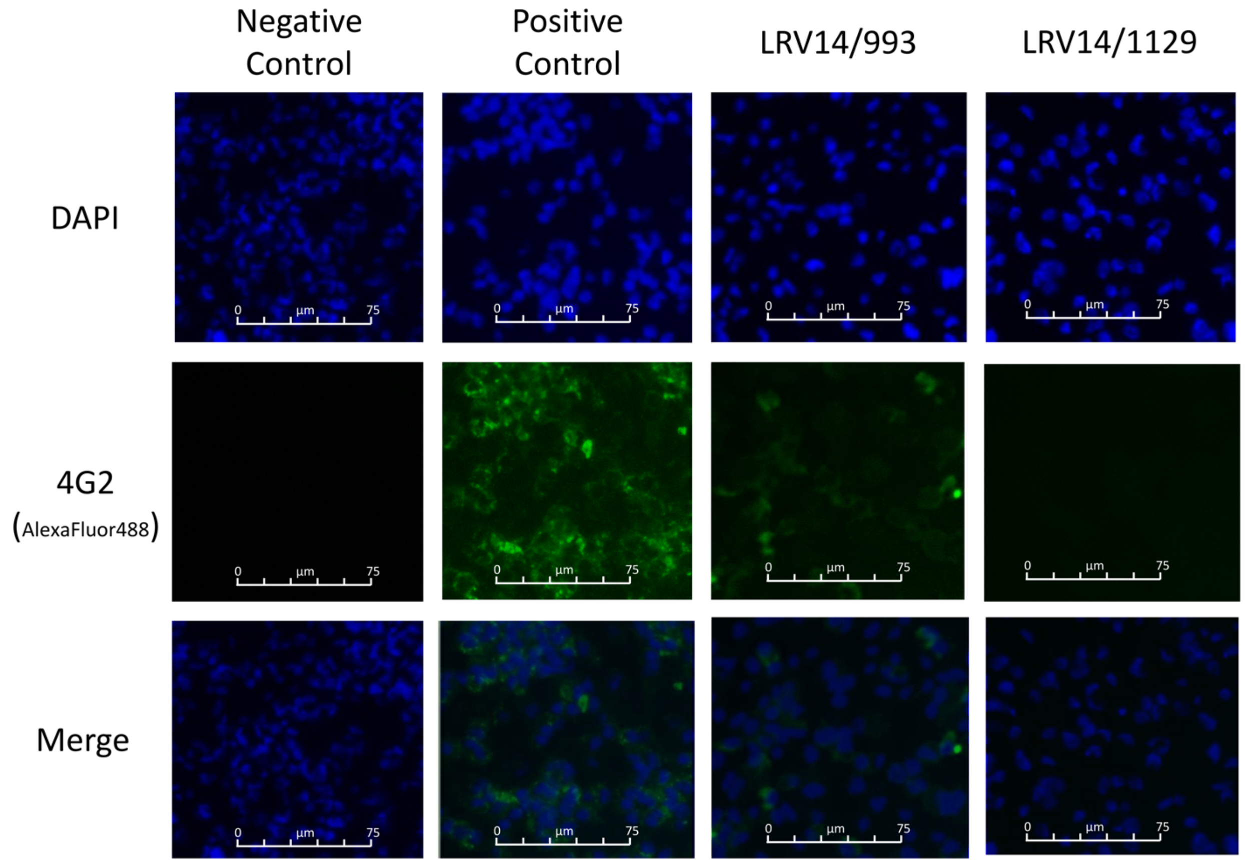

Supplementary Figure S1: Representative images of immunofluorescence on C6/36 cells to confirm dengue virus diagnosis in serum samples. Representative immunofluorescence assay of virus isolation on C6/36 with serum samples from dengue patients (LR14/1129 and LRV14/993). A mock infection was used as a negative control, and infection with DENV-4/TVP/360 MOI 0,1 was used as a positive control. After 7–14 days (depending on the cytopathic effect), the staining was made using anti-E flavivirus monoclonal antibody (4G2) followed by an anti-mouse conjugated with AlexaFluor488 and DAPI. Cells were then observed in an immunofluorescence microscope Nikon Eclipse 80i. The scale bar corresponds to 75 μm.

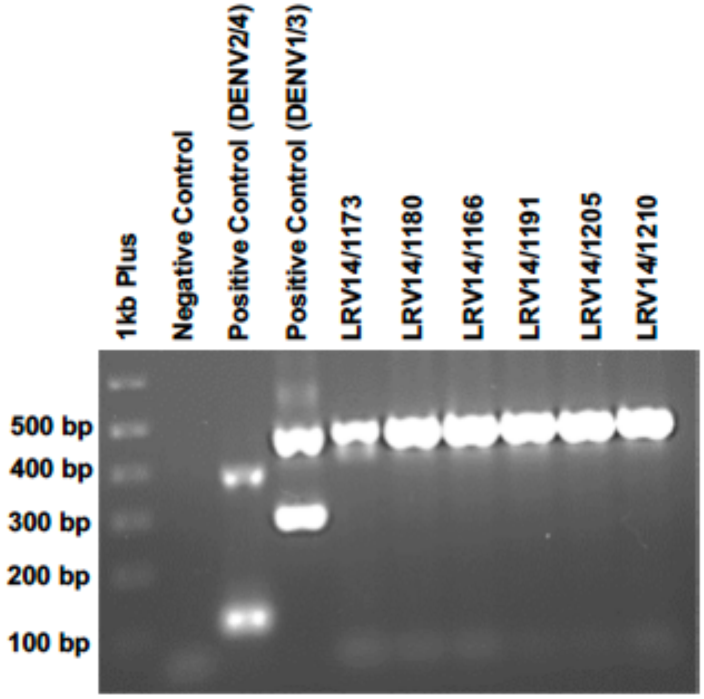

Supplementary Figure S2: Representative agarose gel of one-step RT-PCR to confirm dengue virus diagnosis/serotype in serum samples. After the virus isolation on C6/36, the cell culture supernatant of samples positive by immunofluorescence assays was tested by one-step RT-PCR for DENV serotyping. A 1% agarose gel stained with ethidium bromide showing the serotyping of DENV samples (LRV14/1173, LRV14/1180, LRV14/1166, LRV14/1191, LRV14/1205 and LRV14/1210). A mock control and mixture of RNAs from DENV1+3 (DENV-1/BR/90 and DENV3/BR/97-04) and DENV2+4 (DENV-2/BR/01-01 and DENV-4/TVP/360) were also included. Expected amplicons were 482 bp for DENV-1, 119 bp for DENV-2, 290 bp for DENV3, and 392 bp for DENV-4. A 1Kb plus ladder was also included as a control (ThermoFischerScientific©).

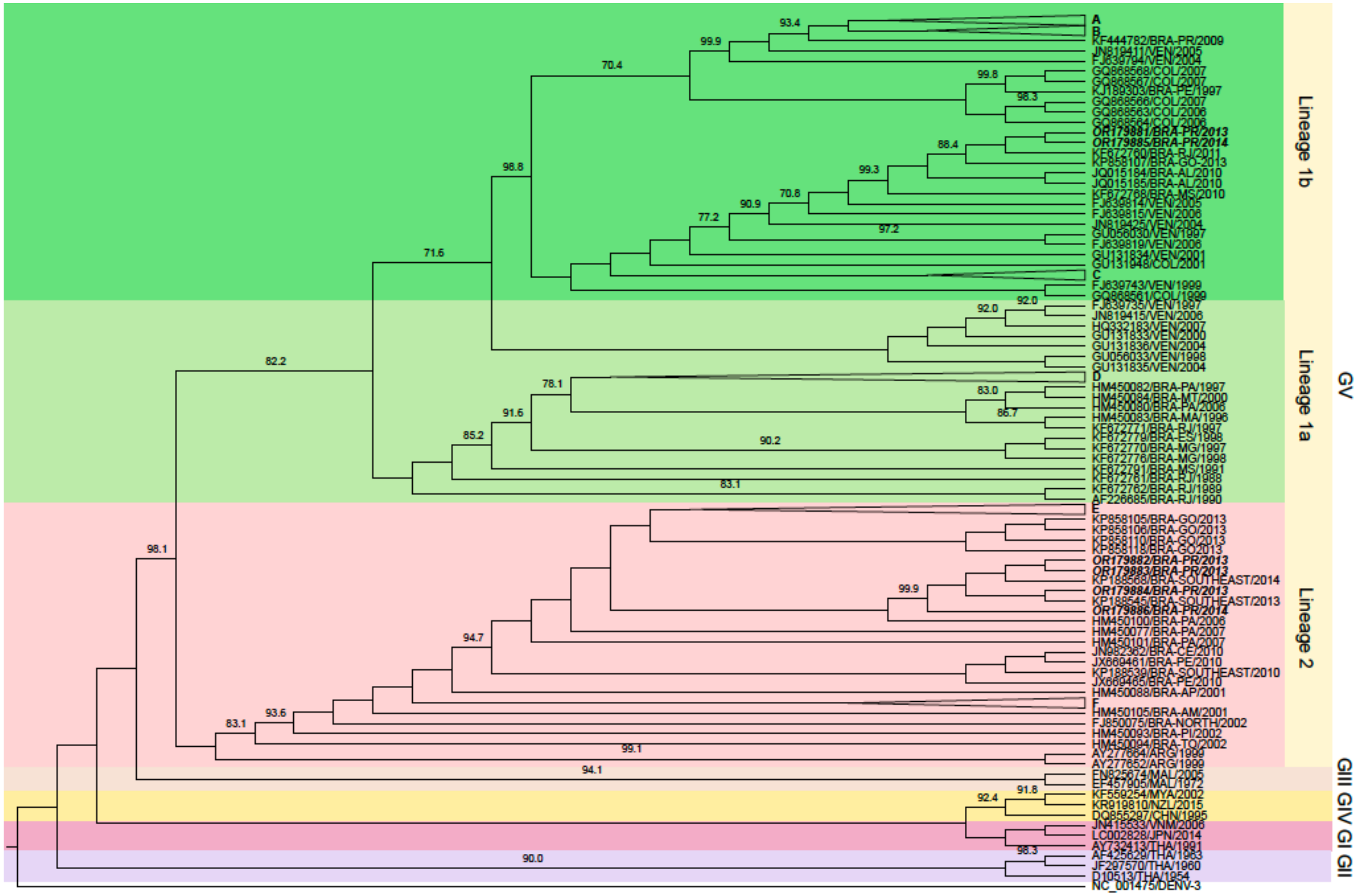

Supplementary Figure S3: Phylogenetic analysis of the E coding region of *Dengue virus* strains from Cambé. The phylogenetic tree was inferred through maximum likelihood using PhyML 3.0, with a GTR + G + I model. Left to the nodes are represented the bootstrap values >70 (1000 replicates). The tree was rooted in an external DENV-3 group (NC_001475), and the tree branch length does not represent the evolutionary distance between groups. Some clades with support inferior to 10 were collapsed for better image resolution, hiding 88 strains (see Supplementary Table 1). Samples sequenced in the present work are highlighted in bold italics. Strains were nominated according to the GenBank (accession number/country/year).

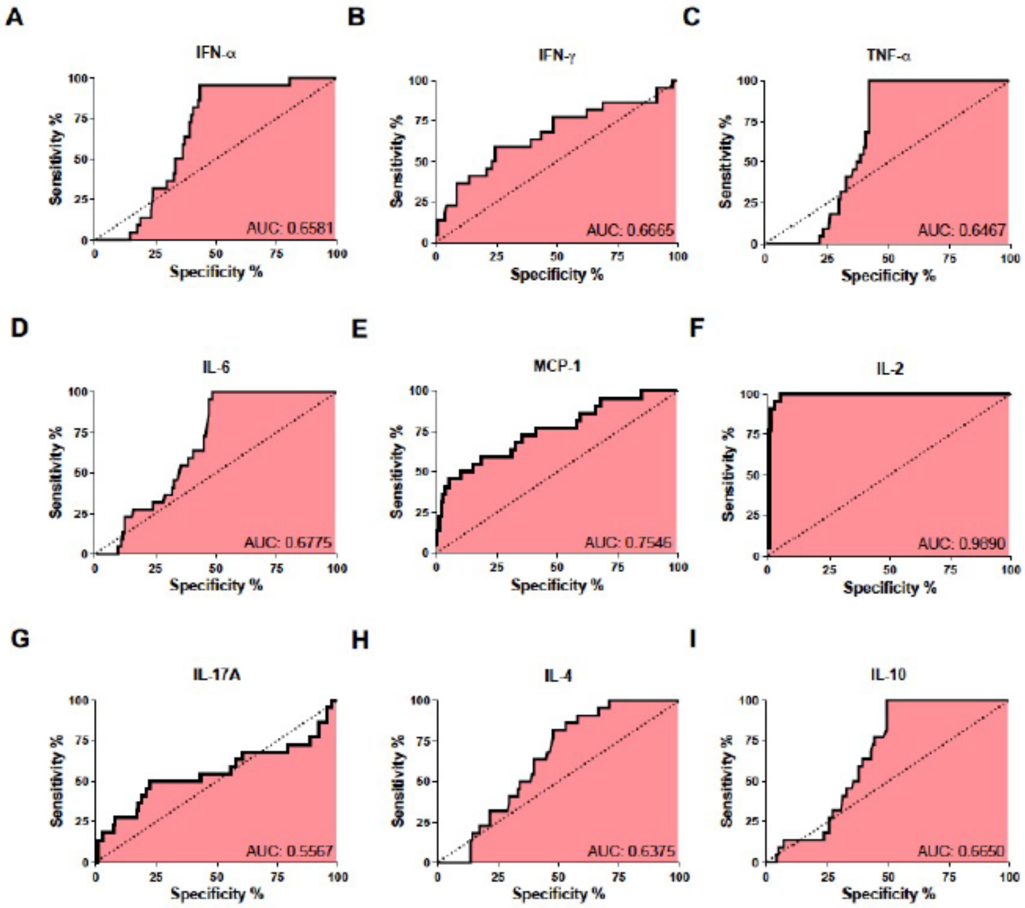

Supplementary Figure S4. Area under the curve (AUC) analysis of cytokines/chemokines in acute phase dengue patients. AUC for cytokines/chemokine IFN-α **(A)**, IFN-γ **(B)**, TNF-α **(C)**, IL-6 **(D)**, MCP-1 **(E)**, IL-2 **(F)**, IL-17A **(G)**, IL-4 **(H)** and IL-10 **(I).** The solid line represents the dots used to build the AUC curve, and the dashed lines represent the identity between specificity and sensitivity. AUC values are depicted in each graph.

Supplementary Table S1:

Name	NCBI number	Country	City/Region	Year	Clade
JN713897_BR_RR_2010	JN713897	Brazil	Roraima	2010	A
KF444786/BRA-RR/2010	KF444786	Brazil	Roraima	2010	A
KF444785/BRA-RR/2009	KF444785	Brazil	Roraima	2009	A
KF444780/BRA-RR/2009	KF444780	Brazil	Roraima	2009	A
HM450104/BRA-RR/2008	HM450104	Brazil	Roraima	2008	A
KF444787/BRA-RR/2010	KF444787	Brazil	Roraima	2010	A
KF444792/BRA-RR/2010	KF444792	Brazil	Roraima	2010	A
KF444790/BRA-RR/2009	KF444790	Brazil	Roraima	2009	A
KF444784/BRA-RR/2009	KF444784	Brazil	Roraima	2009	B
KF444788/BRA-RR/2010	KF444788	Brazil	Roraima	2010	B
KF444791/BRA-RR/2009	KF444791	Brazil	Roraima	2009	B
KF444783/BRA-RR/2009	KF444783	Brazil	Roraima	2009	B
KF444789/BRA-RR/2008	KF444789	Brazil	Roraima	2008	B
FJ639740/VEN/1998	FJ639740	Venezu-ela	Aragua	1998	C
FJ850104/VEN/2008	FJ850104	Venezu-ela	Aragua	2008	C
GU131837/VEN/2005	GU131837	Venezu-ela	Aragua	2005	C
GQ868560/COL/1998	GQ868560	Colombia	Santander	1998	C
GQ868560/COL/1998	GQ868559	Colombia	Santander	1998	C
AF425634/VEN/1997	AF425634	Venezu-ela	Aragua	1997	C
HM450079/BRA-CE/1994	HM450079	Brazil	Ceará	1994	D
JX669469/BRA-PE/1997	JX669469	Brazil	Pernambuco	1997	D
AF311956/BRA-PE/1997	AF311956	Brazil	Pernambuco	1997	D
KF672777/BRA-MG/1998	KF672777	Brazil	Minas Gerais	1998	D
KF672792/BRA-RJ/1999	KF672792	Brazil	Rio de Janeiro	1999	D
KF672763/BRA-ES/2000	KF672763	Brazil	Espírito Santo	2000	D
KF672782/BRA-RJ/2000	KF672782	Brazil	Rio de Janeiro	2000	D
HM450098/BRA-RN/2001	HM450098	Brazil	Rio Grande do Norte	2001	D
KF672783/BRA-ES/2002	KF672783	Brazil	Espírito Santo	2002	D
KF672778/BRA-ES/1998	KF672778	Brazil	Espírito Santo	1998	D
HM450090/BRA-MT/2002	HM450090	Brazil	Mato Grosso	2002	D
HM450091/BRA-MT/2002	HM450091	Brazil	Mato Grosso	2002	D
AY277658/ARG/2000	AY277658	Argentina	Buenos Aires	2000	D
JX669471/BRA/1999	JX669471	Brazil	Pernambuco	1999	D
KF672764/BRA-RJ/2001	KF672764	Brazil	Rio de Janeiro	2001	D
KF672788/BRA-RJ/2001	KF672788	Brazil	Rio de Janeiro	2001	D
HM450086/BRA-CE/2000	HM450086	Brazil	Ceará	2000	D

JX669475/BRA-PE/2002	JX669475	Brazil	Pernambuco	2002	D
KF672773/BRA-RN/1997	KF672773	Brazil	Rio Grande do Norte	1997	D
KF672775/BRA-CE/1998	KF672775	Brazil	Ceará	1998	D
KF672769/BRA-MG/1997	KF672769	Brazil	Minas Gerais	1997	D
KF672774/BRA-RJ/1998	KF672774	Brazil	Rio de Janeiro	1998	D
JX669468/BRA-PE/1997	JX669468	Brazil	Pernambuco	1997	D
KF672772/BRA-RJ/1997	KF672772	Brazil	Rio de Janeiro	1997	D
AF311958/BRA-PE/1997	AF311958	Brazil	Pernambuco	1997	D
AB519681/BRA-DF/2001	AB519681	Brazil	Distrito Federal	2001	D
AF513110/BRA-PR/2001	AF513110	Brazil	Paraná	2001	D
KF672787/BRA-PI/2000	KF672787	Brazil	Piauí	2000	D
JX669474/BRA-PE/2001	JX669474	Brazil	Pernambuco	2001	D
JX669473/BRA-PE/2001	JX669473	Brazil	Pernambuco	2001	D
JX669472/BRA-PE/2000	JX669472	Brazil	Pernambuco	2000	D
KP858119/BRA-GO/2013	KP858119	Brazil	Goiás	2013	E
KP858114/BRA-GO/2013	KP858114	Brazil	Goiás	2013	E
KC692513/ARG/2010	KC692513	Argentina	Buenos Aires	2010	E
KY369949/BRA-SP/2013	KY369949	Brazil	São Paulo	2013	E
KC692511/ARG/2010	KC692511	Argentina	Buenos Aires	2010	E
GU131863/BRA-SP/2008	GU131863	Brazil	São Paulo	2008	E
KP188567/BRA-SP/2013	KP188567	Brazil	São Paulo	2013	E
KP858113/BRA-GO/2013	KP858113	Brazil	Goiás	2013	E
KP858112/BRA-GO/2013	KP858112	Brazil	Goiás	2013	E
KP858117/BRA-GO/2013	KP858117	Brazil	Goiás	2013	E
KP858115/BRA-GO/2013	KP858115	Brazil	Goiás	2013	E
KP858109/BRA-GO/2013	KP858109	Brazil	Goiás	2013	E
JX669464/BRA-PE/2010	JX669464	Brazil	Pernambuco	2010	E
KF672785/BRA-ES/2010	KF672785	Brazil	Espírito Santo	2010	E
KF672759/BRA-RJ/2010	KF672759	Brazil	Rio de Janeiro	2010	E
KP858116/BRA-GO/2013	KP858116	Brazil	Goiás	2013	E
KP858111/BRA-GO/2013	KP858111	Brazil	Goiás	2013	E
KP188544/BRA-SP/2012	KP188544	Brazil	São Paulo	2012	E
KP858108/BRA-GO/2013	KP858108	Brazil	Goiás	2013	E
KP858105/BRA-GO/2013	KP858105	Brazil	Goiás	2013	E
KP858110/BRA-GO/2013	KP858110	Brazil	Goiás	2013	E
KP858118/BRA-GO/2013	KP858118	Brazil	Goiás	2013	E
JN982362/BRA-CE/2010	JN982362	Brazil	Ceará	2010	F
JX669461/BRA-PE/2010	JX669461	Brazil	Pernambuco	2010	F
KP188539/BRA-SP/2010	KP188539	Brazil	São Paulo	2010	F
JX669465/BRA-PE/2010	JX669465	Brazil	Pernambuco	2010	F
HM450088/BRA-AP/2001	HM450088	Brazil	Amapá	2001	F

HM450089/BRA-RR/2001	HM450089	Brazil	Roraima	2001	F
HM450095/BRA-AM/2002	HM450095	Brazil	Amazonas	2002	F
KF672789/BRA-ES/2001	KF672789	Brazil	Espírito Santo	2001	F
FJ850071/BRA-NORTH/2000	FJ850071	Brazil	Norte	2000	F
HM450096/BRA-MA/2003	HM450096	Brazil	Maranhão	2003	F
FJ850084/BRA-NORTH/2005	FJ850084	Brazil	Norte	2005	F
HM450099/BRA-AP/2005	HM450099	Brazil	Amapá	2005	F
FJ850070/BRA-NORTH/2000	FJ850070	Brazil	Norte	2000	F
FJ850081/BRA-NORTH/2004	FJ850081	Brazil	Norte	2000	F
HM450092/BRA-PI/2002	HM450092	Brazil	Piauí	2002	F

Supplementary Table S2: Correlation between viremia and cytokine/chemokine levels in the serum of DENV-infected patients.

**Cytokines/Chemokines**	**Spearman (r)**	**95% confidence interval**	***p-value***
IFN-α	0.1638	-0.008386 to 0.3266	0.0548
IFN-γ	0.01355	-0.1588 to 0.1851	0.8747
TNF-α	0.1474	-0.02523 to 0.3115	0.0845
IL-2	0.01459	-0.1578 to 0.1861	0.8651
IL-6	0.1128	-0.06034 to 0.2794	0.1877
IL-4	0.1603	-0.1204 to 0.3234	0.0604
IL-10	-0.07533	-0.2442 to 0.09793	0.3799
IL-17A	0.04168	-0.1312 to 0.2122	0.6274
MCP-1	0.09741	-0.07585 to 0.2650	0.2557
